# Small global effect on terrestrial net primary production due to increased fossil fuel aerosol emissions from East Asia since the turn of the century

**DOI:** 10.1002/2016GL068965

**Published:** 2016-08-14

**Authors:** M. O'Sullivan, A. Rap, C. L. Reddington, D. V. Spracklen, M. Gloor, W. Buermann

**Affiliations:** ^1^ Institute for Climate and Atmospheric Science, School of Earth and EnvironmentUniversity of LeedsLeedsUK; ^2^ School of GeographyUniversity of LeedsLeedsUK

**Keywords:** fossil fuel aerosol emission, East Asia, aerosol optical depth, diffuse radiation, net primary production

## Abstract

The global terrestrial carbon sink has increased since the start of this century at a time of growing carbon emissions from fossil fuel burning. Here we test the hypothesis that increases in atmospheric aerosols from fossil fuel burning enhanced the diffuse light fraction and the efficiency of plant carbon uptake. Using a combination of models, we estimate that at global scale changes in light regimes from fossil fuel aerosol emissions had only a small negative effect on the increase in terrestrial net primary production over the period 1998–2010. Hereby, the substantial increases in fossil fuel aerosol emissions and plant carbon uptake over East Asia were effectively canceled by opposing trends across Europe and North America. This suggests that if the recent increase in the land carbon sink would be causally linked to fossil fuel emissions, it is unlikely via the effect of aerosols but due to other factors such as nitrogen deposition or nitrogen‐carbon interactions.

## Key Points

- Increase in FF aerosol emission over East Asia contributes substantially to regional increase in plant carbon uptake
- The global impact is small since increases in FF aerosols and NPP in East Asia were canceled by opposing trends in Europe and North America
- Thus, the increase in FF aerosols over East Asia may not act as a mechanism that would explain coincident increase in land carbon sink

## 1 Introduction

Fossil fuel (FF) emissions of CO_2_ have sharply increased since the turn of the century at a rate of 3% yr^−1^, almost twice the rate of the prior three decades [*Hansen et al*., 2013]. In contrast, global atmospheric CO_2_ growth rates were relatively constant during this period [*Ballantyne et al*., 2012]. A coincident decline in land use carbon emissions [*Harris et al*., 2012] as well as a moderate strengthening of ocean carbon uptake [*Rödenbeck et al*., 2014; *Le Quéré et al*., 2015] may have played a role, but these contributions appear insufficient to explain the slow atmospheric growth rate of CO_2_, implying that terrestrial carbon sinks must have substantially increased in this period [*Sarmiento et al*., 2010].

The recent divergence of trends in carbon emissions and atmospheric CO_2_ growth rates led to speculations that key carbon sink processes may be strongly controlled by the increasing emissions themselves, namely, increased nitrogen deposition and a larger fraction of diffuse versus direct solar radiation from predominantly increased sulfate aerosol emissions originating from East Asia [*Hansen et al*., 2013]. In regard to the latter, multiple studies have shown that the efficiency of plant photosynthesis increases under more diffuse light conditions (e.g., resulting from increased scattering of light by aerosols or clouds) since under such conditions radiation can penetrate deeper into the canopy, illuminating previously shaded leaves [*Roderick et al*., 2001; *Gu et al*., 2003; *Mercado et al*., 2009]. However, these studies also show that a corresponding reduction in total radiation may have a negative impact upon photosynthesis, whereby gross primary productivity (GPP) tends to decline if the diffuse fraction surpasses 0.4 [*Mercado et al*., 2009]. The overall effect on photosynthesis and net primary production (NPP) thus depends upon the balance between these two mechanisms. Recent model results showed that increases in the fraction of diffuse radiation due to anthropogenic aerosols in the period 1960–1999 (the global dimming period) enhanced the global carbon sink by 24% [*Mercado et al*., 2009]. The extent at which the rapid increase in East Asian FF aerosol emissions since the turn of the century may have impacted plant growth and the global carbon sink is, however, not clear since anthropogenic aerosol emissions in Europe and United States have decreased persistently since the late 1980s [*Wild et al*., 2009].

Here we therefore test the hypothesis that an increase in the fraction of diffuse light associated with increased FF aerosol emissions predominantly from East Asia has contributed to increased global plant carbon uptake which would provide a mechanism for a potential link between global carbon emissions and the land carbon sink. Using atmospheric models, including an aerosol model with size‐resolved aerosol microphysics, we first simulate aerosol distributions (originating from fossil fuel and fires) and corresponding effects on light regimes over 1998 to 2010. We then use these to drive a land surface model to estimate their relative contributions to changes in regional and global NPP.

## 2 Methodology

The distribution of anthropogenic aerosols was simulated using a global aerosol model [*Mann et al*., 2010]. The impact of aerosols and clouds on surface radiation was simulated using a radiative transfer model [*Edwards and Slingo*, 1996]. Plant carbon uptake was simulated using a land surface model [*Best et al*., 2011; *Clark et al*., 2011]. A similar combination of models has also been used in a recent study by *Rap et al*. [2015].

### 2.1 Aerosol Model

The aerosol distribution was simulated using the Global Model of Aerosol Processes (GLOMAP) [*Mann et al*., 2010], which is an extension to the TOMCAT 3‐D chemical transport model [*Chipperfield*, 2006]. GLOMAP is a global aerosol microphysical model that simulates the concentration, size, and mass of aerosol particles using a two‐moment (mass per particle and number concentration) modal scheme. This model includes various aerosol processes, including nucleation, condensation, growth, coagulation, dry and wet deposition, and cloud processing. In the GLOMAP version used here, the aerosol species included are black carbon (BC), particulate organic matter, sulfate, sea salt, and mineral dust. The horizontal resolution is 2.8° × 2.8°, with 31 vertical levels ranging from the surface to 10 hPa, with the layer thickness varying from 60 m (surface) to 1 km (tropopause). The model is driven with historical meteorology from the European Centre for Medium‐Range Weather Forecasts (ECMWF) at 6‐hourly intervals and interpolated onto the model time step (30 min). Annually varying anthropogenic emissions (BC, organic carbon (OC), and SO_2_) including fossil fuel and biofuel emissions are taken from the MACCity inventory [*Granier et al*., 2011]. This data set is based on historical Atmospheric Chemistry and Climate Model Intercomparison Project (ACCMIP) (for years 1990 and 2000) and RCP 8.5 (2005 and 2010) emissions. The emissions were linearly interpolated for the years between those given. Biomass burning emissions (BC, OC, and SO_2_) are taken from the Global Fire Emissions Database version 3 [*van der Werf et al*., 2010] and are supplied as annually varying monthly means.

**Figure 1:**
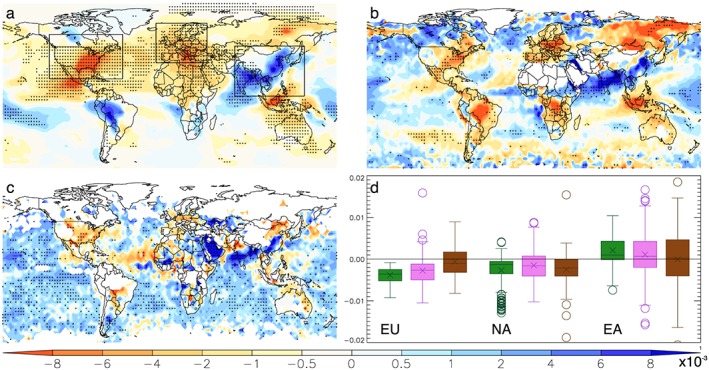
Comparison between modeled and satellite annual mean AOD trends (year^−1^) for the period of overlapping data records 2001–2010. Panels depict linear trends for (a) GLOMAP, (b) MODIS, and (c) SeaWiFS. (d) Linear trends in AOD (year^−1^) between 2001 and 2010 are shown for the three focus regions (outlined in Figure 1a; land points only): Europe (EU), North America (NA), and East Asia (EA) based on GLOMAP (green), MODIS (violet), and SeaWiFS (brown). The crosses represent the mean trend, the middle bars the median, the boxes the 25th and 75th percentile values, and the error bars the minimum and maximum values with circles representing outliers (greater than 1.5 times interquartile range). White areas in Figures 1b and 1c indicate regions where satellite retrievals were not available, and in all maps statistically significant (*P* < 0.05; Student's *t* test) trends are highlighted with stippling. Spatial resolutions in the original data sets differ between modeled (2.8°) and satellite (MODIS (1.0°) and SeaWiFS (0.5°)), and for this comparison the satellite AOD fields were aggregated to the coarser model resolution.

GLOMAP has been evaluated extensively in previous work and generally found to match ground‐based station observations (e.g., Aerosol Robotic Network) well [*Mann et al*., 2010; *Reddington et al*., 2014, 2016; *Rap et al*., 2015]. In this study, we compared trends in simulated aerosol optical depth (AOD) with satellite‐based (Moderate Resolution Imaging Spectroradiometer (MODIS) and Sea‐viewing Wide Field‐of‐view Sensor (SeaWiFS)) [*Hsu et al*., 2013; *Platnick et al*., 2015] estimates for the period of overlapping data records 2001–2010. Results showed that while there is generally good agreement between the modeled and observed AOD trends in areas where fossil fuel emissions dominate the AOD pattern which is the focus of this study (Figure 1 and Figures S1–S3 in the supporting information), there are also notable differences in specific regions (e.g., Amazon Basin). Some reasons for these discrepancies may involve the comparatively larger interannual variability in the satellite AOD (Figure S2), requiring greater changes to be significant. In addition, the GLOMAP “baseline” AOD magnitudes tend to be somewhat lower than the satellite AOD (Figure S2); therefore, trends of equal size are more likely to be significant in the simulated AOD. In this study we are interested in trends in AOD driven by changing anthropogenic aerosol emissions. To exclude a contamination from dust, we calculate AOD only for the four aerosol size modes (aitken soluble, aitken insoluble, accumulation soluble, and coarse soluble) that do not include dust. We demonstrated that satellite aerosol trends are similar during periods both with and without a large contribution from dust in East Asia (Figure S1), demonstrating that observed trends are not due to trends in dust.

### 2.2 Radiative Transfer Model

The *Edwards and Slingo* [1996] radiative transfer model is used to quantify the aerosol effect on direct and diffuse radiation [*Rap et al*., 2013]. We used the aerosol optical properties (scattering, absorption, and asymmetry coefficients) for each aerosol mode and spectral band based on *Bellouin et al*. [2013]. The model is forced with monthly mean ECMWF climate (water vapor and temperature) and ozone reanalysis data together with cloud fields and surface albedo from the International Satellite Cloud Climatology Project (ISCCP‐D2) [*Rossow and Schiffer*, 1999]. The simulated total and direct radiation fluxes are used to calculate diffuse radiation (diffuse = total − direct). Due to the uncertainty in aerosol‐cloud interactions, we do not allow changes in aerosol to alter cloud properties (aerosol indirect effect). The Edwards‐Slingo (ES) model has been validated in recent studies to some extent [e.g., *Rap et al*., 2015]. We performed additional validations at four FluxNet (La Thuile 'fair use' database; http://www.fluxdata.org) sites in Europe and North America and also found generally good agreement between observed and modeled light regimes; however, at some of the sites overestimation of total radiation and underestimation of diffuse radiation were apparent (Figures S4 and S5). This may lead to an overestimation of the diffuse effect on NPP due to the strong nonlinear dependence of plant carbon uptake to changes in diffusivity [*Mercado et al*., 2009].

### 2.3 Land Surface Model

The Joint UK Land Environment Simulator (JULES) land surface model used here simulates the exchange of carbon, water, energy, and momentum between the land surface and atmosphere [*Best et al*., 2011; *Clark et al*., 2011]. The model includes a multilayer (10 levels) canopy parameterization to scale photosynthesis from leaf to the canopy [*Mercado et al*., 2007, 2009]. Photosynthesis is calculated at each level and treats sunlit and shaded leaves separately. In our simulations, we used the dynamic phenology (TRIFFID) version of JULES. To ensure that the plant pools and NPP are at steady state, the model was spun‐up for 60 years (10 in equilibrium mode and 50 in dynamical mode [see *Cox*, 2001]) using a repeated driver climatology for 1995. The control simulation was then run with transient driving input for 1996–1998, providing a steady state to start our simulations from. The model is forced with ERA‐Interim climate fields [*Weedon et al*., 2014] and runs at 0.5° spatial resolution with 3‐hourly time steps. The climate drivers consist of 2 m air temperature, specific humidity, precipitation, 10 m wind speed, and surface pressure. Model drivers also include downward surface radiation (short‐wave direct and diffuse and long wave) from the ES model. The JULES plant carbon uptake response to changes in solar radiation has also been validated to some extent at temperate needleleaf and broadleaf forest sites [*Mercado et al*., 2009] and in tropical rainforests [*Rap et al*., 2015]. We conducted further validations at the same four FluxNet sites that were used in the ES validations (see above). Also in this case, the modeled GPP responses to increases in photosynthetically active radiation under both total and diffuse light regimes agree generally well with observed responses (Figure S6).

We performed a set of factorial simulations with JULES over the period 1998–2010 to isolate the impact of single drivers on NPP. The five drivers considered include (1) climate, (2) atmospheric CO_2_, and incoming solar radiation due to aerosols associated with (3) anthropogenic emissions, (4) fire emissions, and (5) cloud cover. We started with a “control” simulation in which only climate variables were varied and anthropogenic and fire aerosol emissions remained at year 2000 values to avoid the anomalous 1998 El Niño–Southern Oscillation year, and atmospheric CO_2_ was held fixed at 1998 levels, whereas cloud cover was based on a climatology for the whole study period 1998–2010. Four additional simulations were carried out whereby in each simulation one additional driver was varied, so that our final simulation had monthly varying fire emissions and cloud cover for the whole period and anthropogenic emissions and the atmospheric CO_2_ level varied annually. We first calculated the trend (based on linear regression) in annual AOD, surface diffuse radiation (SDR), and NPP for each simulation. The climate effect and combined effect can be inferred directly from the first (only climate varied) and last (all drivers varied) model runs. To isolate the impact of the remaining single drivers, the difference between the trends of two simulations that only differ by that driver was used.

## 3 Results

**Figure 2:**
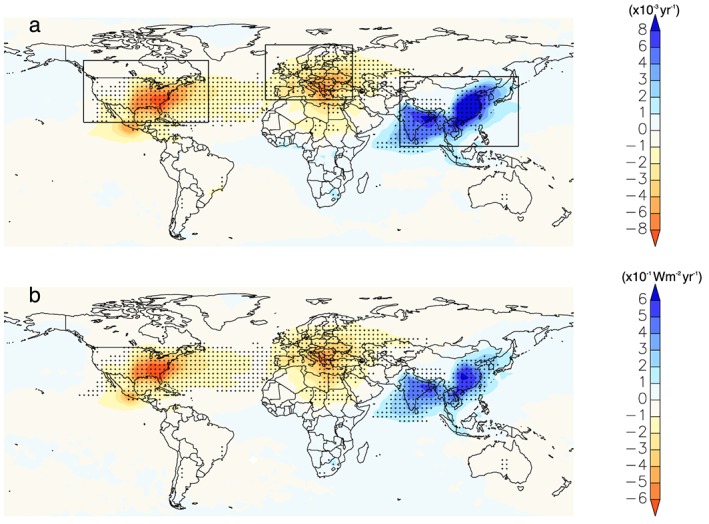
Spatial pattern of linear trends in simulated annual (a) AOD and (b) SDR due to changes in fossil fuel aerosol emissions over the period 1998–2010. In Figures 2a and 2b, trends are calculated as the difference in the trends based on two single simulations, with varying anthropogenic aerosol emissions as the only difference between the two (see section 2). Statistically significant (*P* < 0.05) trends are highlighted with stippling.

The simulated impact of anthropogenic aerosol emissions on AOD and SDR from 1998 to 2010 is shown in Figure 2. As anticipated, AOD changes were largest in regions of significant FF aerosol emission change over this period. For example, East Asia shows substantial increases in AOD and SDR coinciding with increasing anthropogenic aerosol and aerosol precursor emissions [*Granier et al*., 2011]. In contrast, Europe and North America experienced declining AOD and SDR trends driven by a reduction in FF aerosol emissions (Figure 2, Table 1, and Figure S3). The spatial distribution of these trends in AOD and SDR is greatest close to the vicinity of the respective source regions, although changes extend for thousands of kilometers due to atmospheric transport of the aerosols. Our results also show that changes in fossil fuel aerosol emissions play an important role in the AOD trends compared to natural (e.g., sea spray) and fire‐induced changes in all three regions of interest (Figure S3). A subsequent analysis that isolates the contribution of each factor (FF, fire, and clouds) to trends in SDR further confirms this result, with fossil fuel burning also dominating the trend in the three focus regions of East Asia, Europe, and North America (Figure S7).

**Table 1:** Trends in AOD, SDR, and NPP Over the Period 1998–2010 for Global Land and Three Focus Regions^a^

Region	AOD (Year^−1^)	SDR (W m^−2^ yr^−1^)	NPP (Tg C yr^−2^)
East Asia	0.0037*** (0.0035***)	0.31** (0.21***)	44.13* (14.44)
Europe	−0.0052*** (−0.0050***)	−0.56*** (−0.47***)	19.10 (−8.09)
North America	−0.0021*** (−0.0021***)	−0.16 (−0.18***)	17.32 (−9.78)
Global	−0.0002 (−0.0001)	−0.002 (−0.03)	140.13 (−6.82)

a The linear trends shown are based on simulations in which all drivers are varied and where the effect of FF aerosol emissions is isolated (in parentheses). The three focus regions (only land areas) are outlined in Figure 2a.

* Significant at *P* < 0.05 level.

** Significant at *P* < 0.01 level.

*** Significant at *P* < 0.001 level.

**Figure 3:**
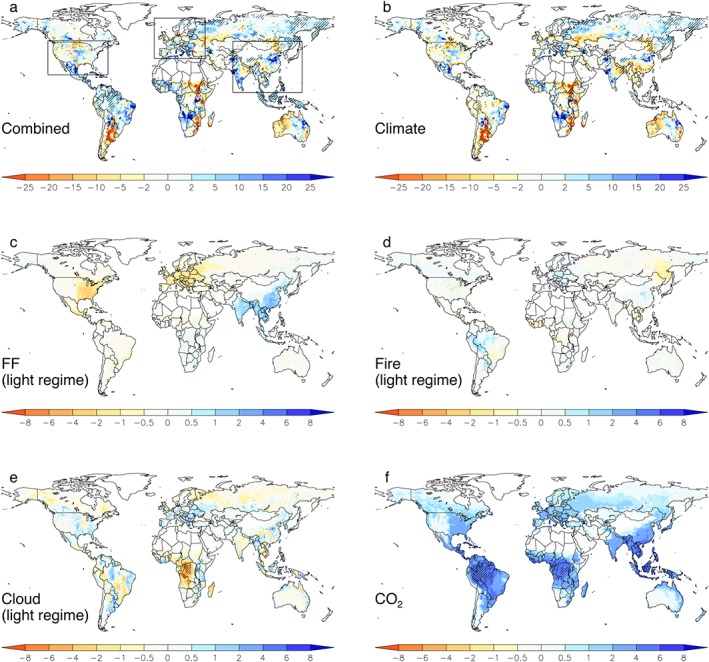
Spatial pattern of linear trends (g C m^−2^ yr^−2^) in annual NPP for the period 1998–2010. The maps depict trends in NPP based on factorial JULES simulations with (a) all drivers varied and corresponding to single drivers including (b) climate, as well as light regimes associated with (c) fossil fuel aerosol emissions, (d) fire aerosol emissions, and (e) cloud cover. (f) Trends in NPP associated with atmospheric CO_2_. Statistically significant (*P* < 0.05) trends are highlighted with stippling.

A factorial analysis based on multiple runs with the JULES land surface model (see section 2) was used to quantify the contribution of single drivers (changes in light regimes due to FF and fire emissions as well as changes in cloud cover, in addition to changes in near‐surface climate and increased atmospheric CO_2_ concentrations) to the trend in NPP in the study period 1998–2010. Results show that the spatial patterns in the overall NPP trends (Figure 3a) were generally dominated by trends in near‐surface climate (Figures 3b and S8). In this regard, warming across northern Eurasia and cooling across Canada appeared to be responsible for the pronounced positive and negative NPP trends in these regions, respectfully (Figures 3b and S9). Over many land regions outside the northern high latitudes, trends in precipitation appeared to be the dominant driver for trends in NPP (Figures 3b and S9).

At more regional levels, changes in SDR associated with FF aerosols had a sizeable impact on trends in NPP in East Asia, Europe, and eastern U.S. (Figure 3c) broadly in line with the spatial pattern of the corresponding AOD and SDR trends (Figure 2). Changes in NPP due to trends in SDR resulting from changes in fire emissions and cloud cover were of similar magnitude but displayed a more heterogeneous pattern across the continents (Figures 3d and 3e). Over the central African rainforests, a relatively strong cloud cover‐SDR effect was observed, where a reduction in SDR associated with a strong trend toward lower cloud cover (Figure S7) led to markedly lower NPP. Conversely, and as expected, the CO_2_ fertilization effect (Figure 3f) led to consistent increases in NPP across most of the vegetated land surface, with the largest impact in the highly productive tropics.

**Figure 4:**
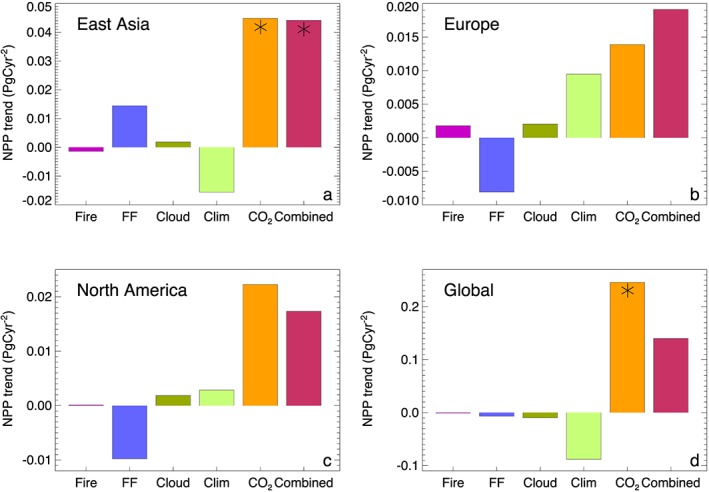
Global, regional, and mechanistic attribution of trends in annual NPP for the period 1998–2010. Trends are based on annual means of spatially aggregated NPP for the three focus regions (a) East Asia, (b) Europe, and (c) North America as well as for (d) all land regions. The three focus regions are depicted in Figure 3a. Statistically significant (*P* < 0.05) trends are highlighted (asterisk).

In Figure 4, regionally aggregated and global contributions from each single driver to the overall NPP trends over the 1998–2010 study period are shown. Corresponding results show that over East Asia, changes in climate (negative contribution) as well as atmospheric CO_2_ (positive) were the most dominant drivers of trends in NPP (Figure 4a). However, increases in SDR due to increasing FF aerosol emissions caused a sizeable positive NPP trend (14 Tg C yr^−2^; see also Table 1), which amounted to a substantial proportion (33%) of the total positive NPP trend over this region. In Europe and North America, changes in climate and atmospheric CO_2_ were generally also the dominant drivers of NPP changes, whereas declining SDR (from decreasing FF aerosol emissions) led to significant negative contributions to the overall NPP trends (Figures 4b and 4c and Table 1). At global scale, we estimated an overall increasing NPP trend of 0.14 Pg C yr^−2^ over the study period 1998–2010 with changes in atmospheric CO_2_ (0.25 Pg C yr^−2^) and near‐surface climate (−0.09 Pg C yr^−2^) playing a dominant role (Figure 4d). At this global level, the aerosol radiative effects from changes in FF emissions are relatively small (−6.8 Tg C yr^−2^, −4.9% of total NPP trend) since the increasing contributions over East Asia are effectively canceled out by the declining contributions from Europe and North America.

## 4 Discussion

Our results suggest that the simulated increase in global NPP (0.14 Pg C yr^−2^) over the period 1998 to 2010 is largely driven by increasing atmospheric CO_2_, through a combination of direct CO_2_ fertilization and the indirect effects of improved water use efficiency in line with previous model studies [*Schimel et al*., 2015; *Sitch et al*., 2015]. The dominant contribution of the CO_2_ fertilization effect on trends in NPP should, however, be viewed with caution as more recent studies showed that land surface models may overestimate corresponding impacts considerably [*Brienen et al*., 2015; *Smith et al*., 2015]. At the global scale, climatic trends over this period contributed negatively to changes in global NPP consistent with results based on a more data‐constrained approach [*Zhao and Running*, 2010].

Radiative effects associated with aerosol emissions from FF and fire activity and those related to clouds on trends in NPP played only a minor role at global scale. Our results, however, do show that at more regional levels, FF aerosol emissions and corresponding effects on diffuse radiation are potent drivers of NPP changes, particularly over East Asia where they contribute 33% to the total NPP trend. In this region, the recent trend in fossil fuel aerosol emissions are mainly driven by increases in coal burning and associated sulfate aerosols [*Lu et al*., 2010; *Granier et al*., 2011]. Our results must be viewed with some caution since, for example, we did not consider potential adverse effects of acidic sulfate deposition on NPP [*Büntgen et al*., 2014] and the effect of diffuse radiation on NPP at regional scales might be slightly overestimated due to a model bias (see section 2.2). But one important inference is that due to the importance of this “FF aerosol driver” and the relatively short atmospheric lifetime of aerosols (days to weeks), a decline in regional‐scale FF aerosols (e.g., through implementing more strict air pollution standards) may reduce NPP and net carbon uptake substantially at relatively short time scales.

Our findings presented here thus indicate that the marked post‐2000 increase in the global land carbon sink may not be explained by changes in light regimes resulting from coincident changes in fossil fuel aerosol emissions and corresponding effects on NPP. This is to a large part a result of the opposing contributions from Asia and from Europe and North America leading to a relatively small global impact. This opens the door for investigations of alternative carbon sink mechanisms that are causally linked to increasing FF emissions. In this regard, nitrogen deposition may act as a potent driver through both its direct effect on photosynthesis, plant respiration, and soil respiration [*Zaehle*, 2013] as well as indirectly through easing nutrient constraints for NPP enhancements via the CO^2^ fertilization effect [*Norby et al*., 2010]. In addition, decadal climatic trends that are largely independent of FF emission trajectories may induce strong impacts on NPP (as shown here) and also on plant and soil respiration. In this regard, the recent “hiatus” in global temperatures [*Intergovernmental Panel on Climate Change*, 2013] may have reduced respiratory carbon fluxes thereby contributing to the enhanced land carbon sink in this time frame.

## Supporting information



Supporting Information S1
