# Supplementary material for: Small global effect on terrestrial net primary production due to increased fossil fuel aerosol emissions from East Asia since the turn of the century
Source: Geophys Res Lett. 2016 Aug 14;43(15):8060–7. doi: 10.1002/2016GL068965 (PMC5053272; doi:10.1002/2016GL068965)
Supplement: Supplementary file 1 — Supporting Information S1 [file GRL-43-8060-s001.pdf]

**Small global effect on terrestrial net primary production due to increased fossil fuel aerosol emissions from East Asia since the turn of the century**

Author list: M. O'Sullivan, A. Rap, C. L. Reddington, D. V. Spracklen, E. Gloor, and W. Buermann

## SUPPORTING INFORMATION

**Figure S1: Comparison between modelled and satellite AOD trends for the period of overlapping data records 2001-2010**

**Figure S2: Time series comparison between modelled and satellite AOD for the period of overlapping data records 2001-2010 in selected regions**

**Figure S3: Spatial pattern of linear trends in simulated annual AOD due to single drivers and the spatial pattern of the dominant driving factor in (GLOMAP) AOD trends over 1998-2010**

**Figure S4: Simulated versus observed monthly mean total and diffuse radiation for four sites in Europe and North America.**

**Figure S5: Simulated and observed total and diffuse radiation seasonal cycle for four sites in Europe and North America.**

**Figure S6: Simulated and observed light response of GPP to direct and diffuse PAR at four forest sites in Europe and North America.**

**Figure S7: Spatial pattern of linear trends in simulated annual SDR due to single drivers and the spatial pattern of the dominant driving factor in SDR trends over 1998-2010 based on the Edwards and Slingo radiative transfer model**

**Figure S8. Spatial pattern of dominant driving factor in simulated (JULES) NPP trends over 1998-2010**

**Figure S9. Spatial distribution of trends in selected land climate drivers for the period 1998-2010**

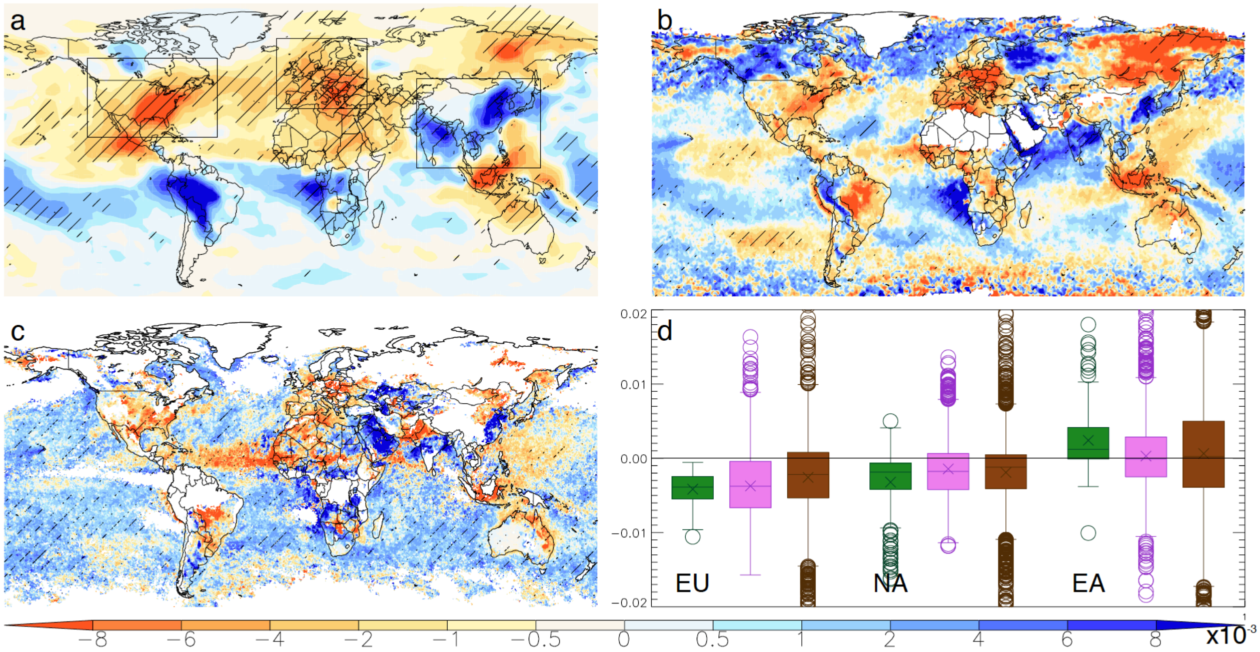


**Figure S1.** Comparison between modelled and satellite AOD trends ($yr^{-1}$) for the period of overlapping data records 2001-2010. Panels depict linear Jul-Dec half-yearly mean trends for (a) GLOMAP, (b) MODIS, and (c) SeaWiFS. In (d), linear trends in AOD ($yr^{-1}$) between 2001 and 2010 are shown for the three focus regions (land points only): Europe (EU), North America (NA), and East Asia (EA) based on GLOMAP (green), MODIS (violet), and SeaWiFS (brown). The crosses represent the mean trend, the middle bars the median, the boxes the $25^{th}$ and $75^{th}$ percentile values and the error bars the minimum and maximum values with circles representing outliers (greater than 1.5 x interquartile range). The three focus regions are depicted in the top left panel. In the maps, white areas in (b) and (c) indicate regions where satellite retrievals were not available and statistically significant ($P<0.05$) trends are highlighted with stippling.

In our GLOMAP AOD calculations we did not consider all aerosol size modes and so miss the majority of dust aerosol, which has potential to cause discrepancies between model and observations. To assess whether dust may have a strong influence on the annual AOD trends (Figure 1 in main manuscript), we also computed AOD trends based on Jul-Dec means since at that time dust is generally not dominating the AOD fields specifically in East Asia (Hansen et al., 2013; ref. in main manuscript). Comparing the (GLOMAP) simulated AOD trends with the satellite observed trends generally showed a good agreement specifically in areas where FF aerosol emissions dominate the AOD trends (see also Figure S1). Further, a comparisons of the satellite AOD trends based on annual and half-yearly means also shows good agreement suggesting that dust does not play a dominant role in the observed AOD trends.

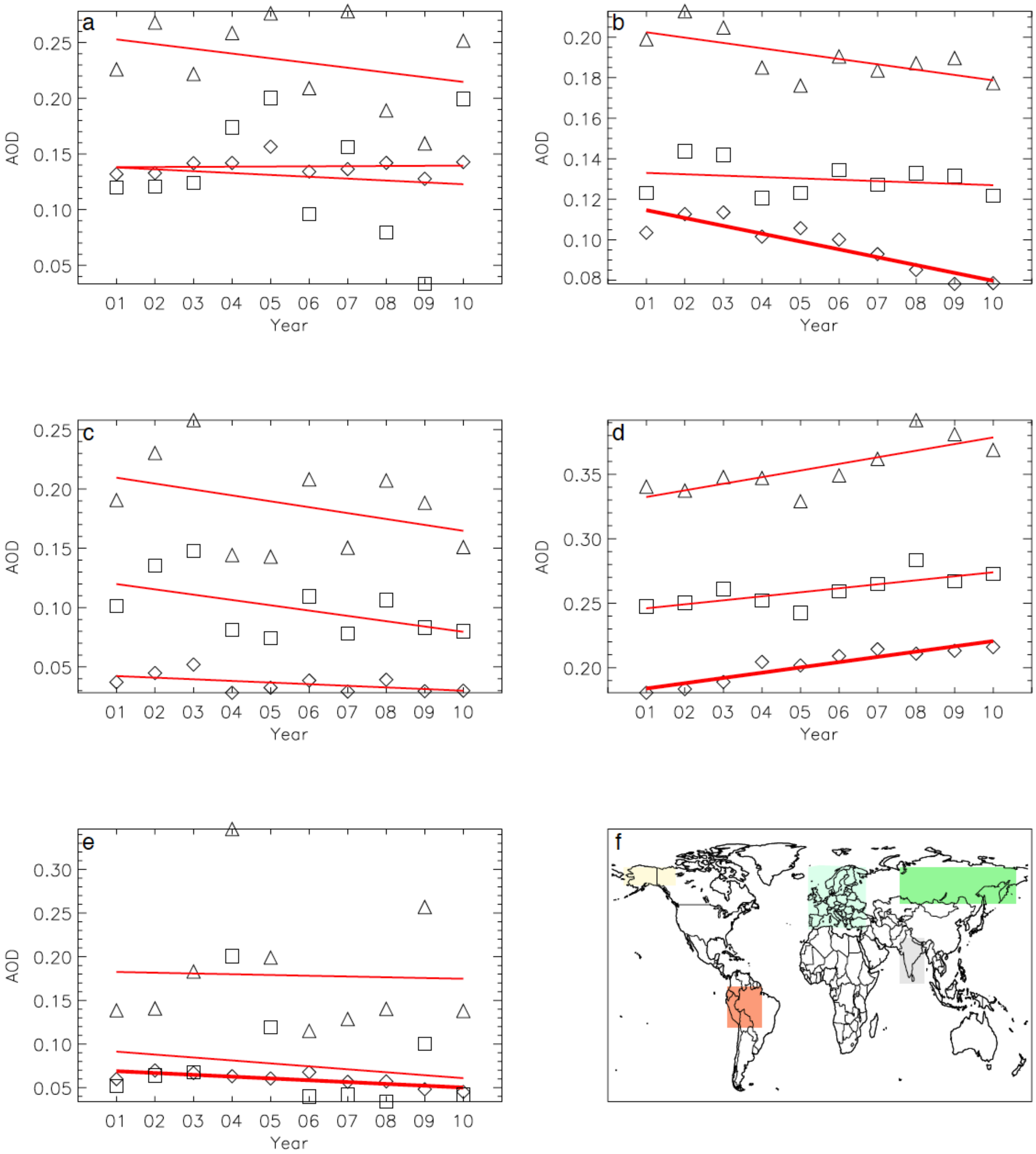


**Figure S2.** Simulated and observed annual mean AOD time series for five regions where discrepancies between modelled and observed AOD trends at some level were identified (see Figure 1 in main ms): a) Amazon, b) Europe, c) north east Russia, d) India, and e) Alaska. Regions are shown in f). Results are shown for GLOMAP (diamonds), SeaWiFS (squares), and MODIS (triangles). Linear best fit lines are plotted, with statistically significant trends (*P*<0.05) bolded.

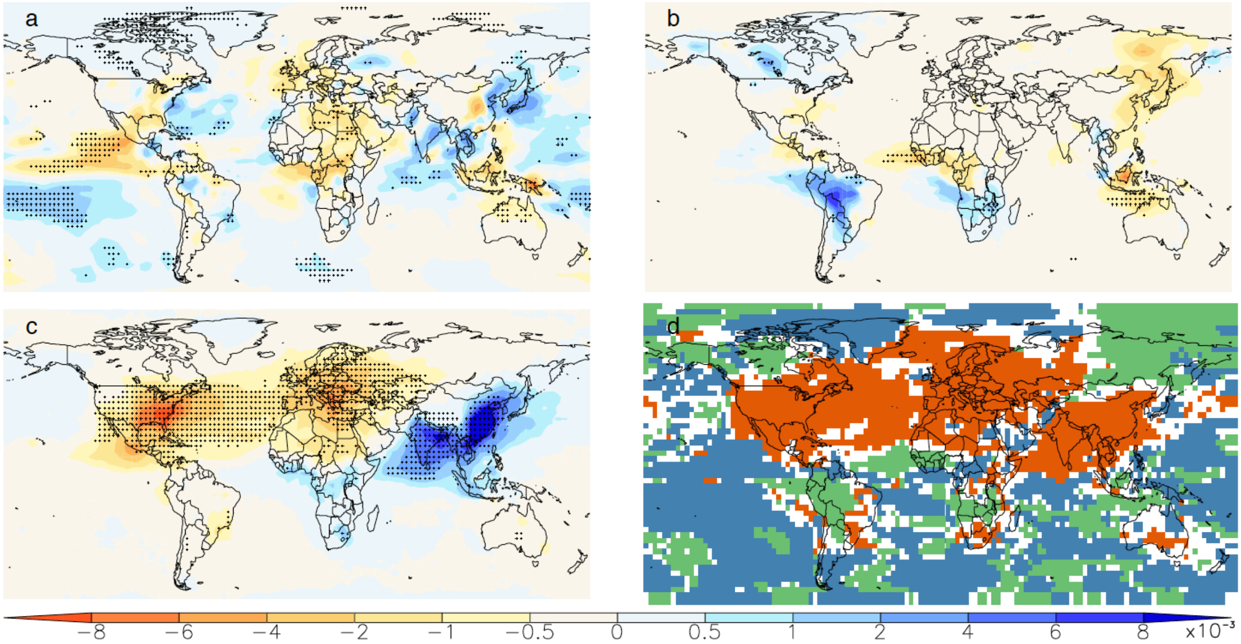


**Figure S3**. Spatial pattern of linear trends ($yr^{-1}$) in simulated annual AOD due to each factor (a-c) and the dominating driving factor in the trend (d) over 1998-2010. The isolated factors include (a) climate, (b) fire emissions, and (c) fossil fuel emissions. Panel (d) shows the dominating driver with climate (blue), fires (green), and fossil fuels (orange) depicted. White areas depict regions with no factor contributing more than 50% to the total trend (no dominating factor).

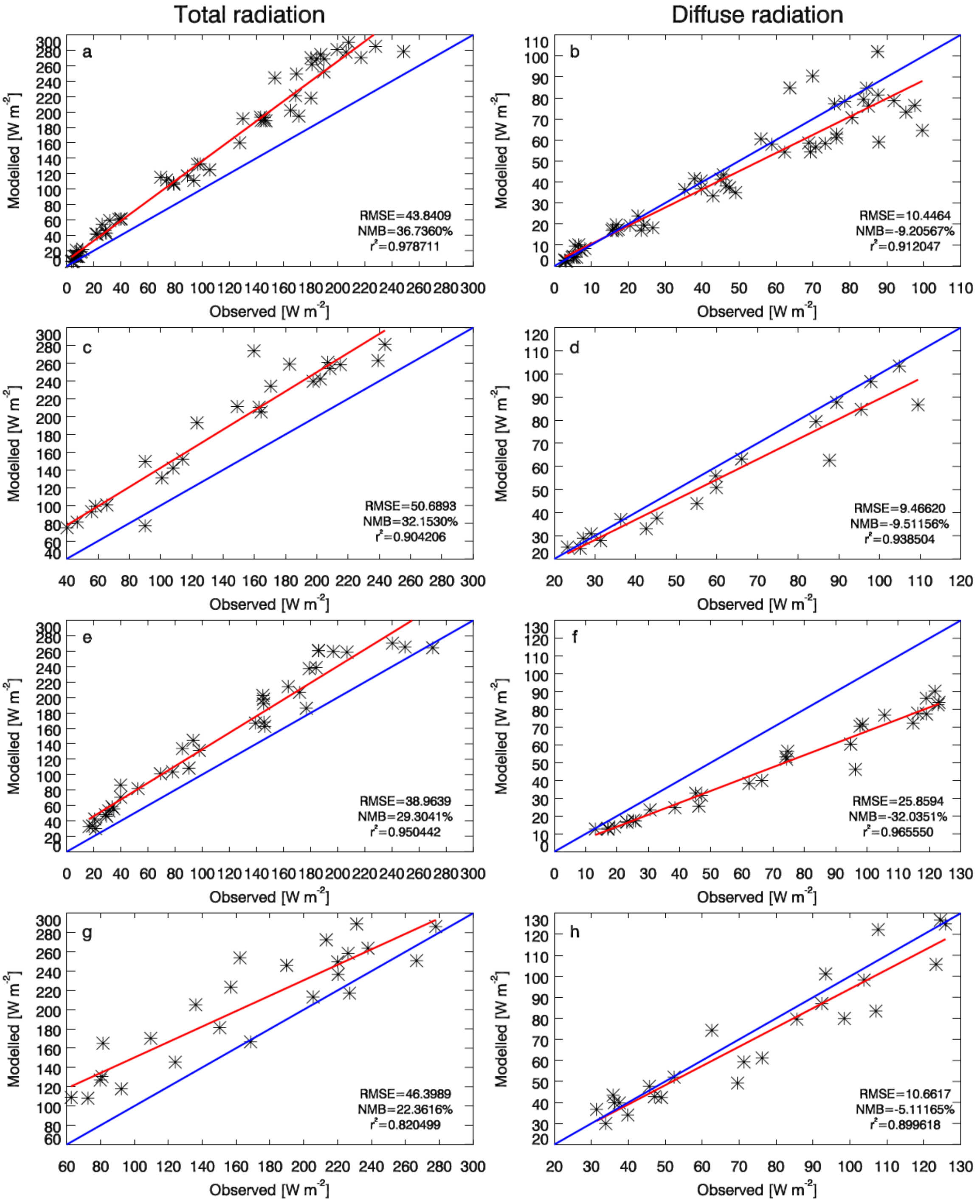


**Figure S4.** Scatterplot of monthly mean observed (FluxNet) versus ES modelled (a,c,e,g) total and (b,d,f,h) diffuse radiation at (a,b) Hyytiala, Finland (Hyy) (61.85° N, 24.30° E), (c,d) Bartlett Experimental Forest, New Hampshire, US (Bar) (44.06° N, 71.29° W), (e,f) Loobos, Netherlands (Loo) (52.17° N, 5.74° E), and (g,h) Walker Branch Watershed, Tennessee, US (WBW) (35.96° N, 84.29° W). The normalised mean bias (NMB), $r^2$ and root-mean-square error (RMSE) between model and observations are shown in each panel. Linear best fit lines are also plotted.

This data model comparison shows generally a good agreement, albeit with high model bias in total radiation (22%<NMB<37%). Simulated diffuse radiation matched the observations also well (-32%<NMB<-5%), with the $r^2$-value between modelled and observed radiation (total and diffuse) being high at all sites ($r^2$>0.82).

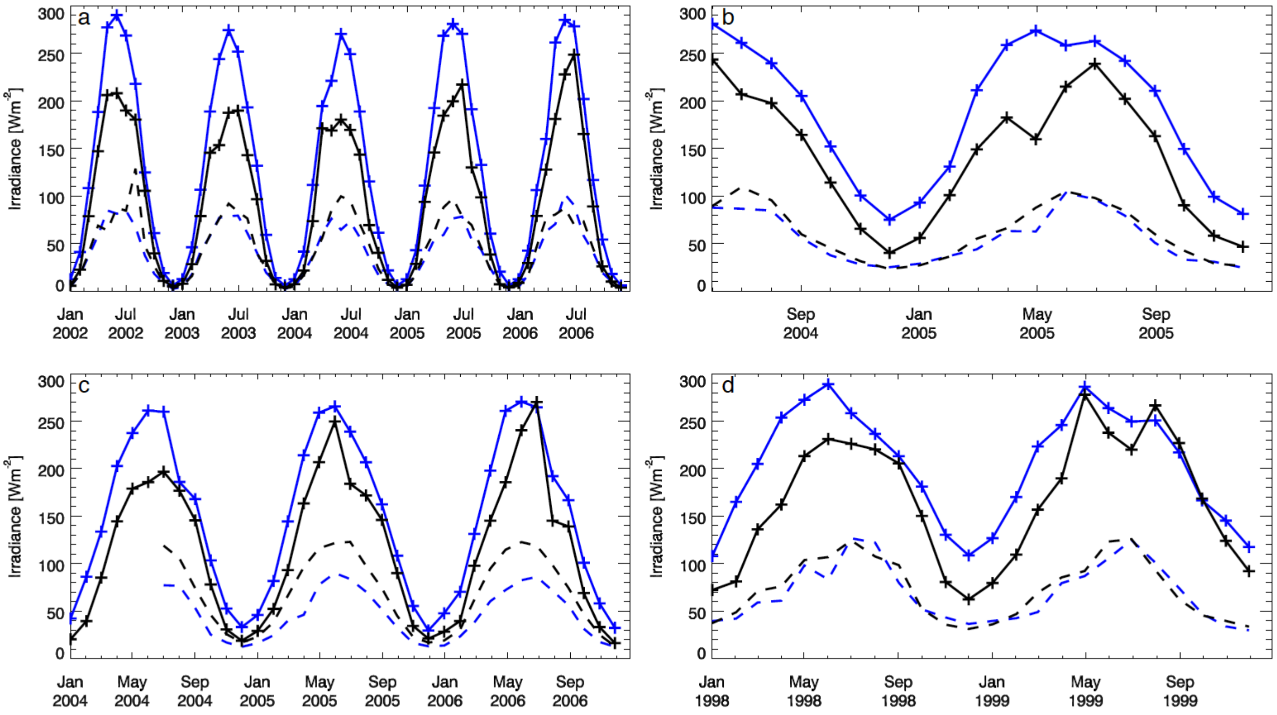


**Figure S5.** Observed (black) and ES modelled (blue) monthly mean total (solid) and diffuse (dashed) radiation at a) Hyy, b) Bar, c) Loo, and d) WBW.  Results show that the model also captures the observed seasonal cycle of surface radiation. The aforementioned high model bias in total radiation (Figure S4) is notable at Hyytiala, however the model performs well at the other three sites, especially in matching observed diffuse radiation trajectories.

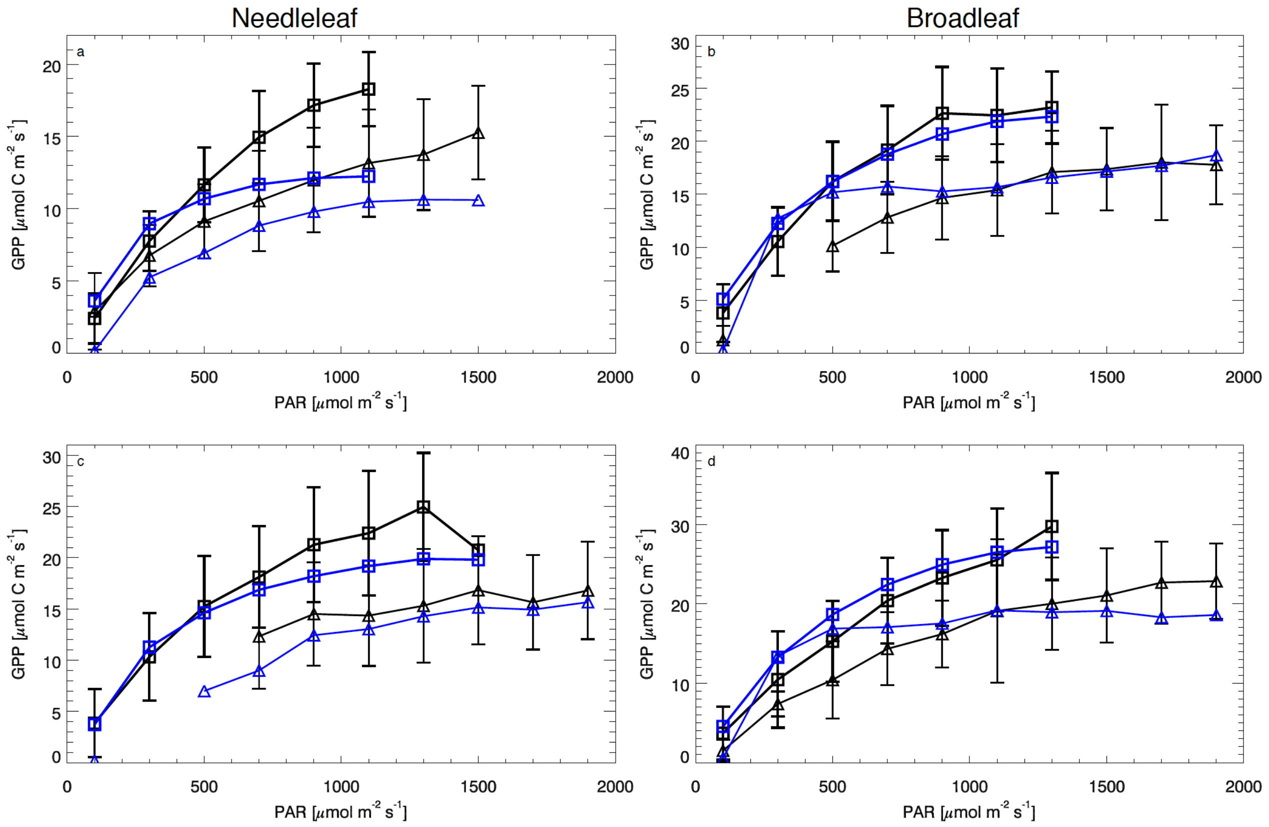


**Figure S6.** Observed (black) and modelled (blue) GPP response to both direct (triangles) and diffuse (squares) photosynthetic active radiation (PAR) averaged for bins of 200 μmol $m^{-2}$ $s^{-1}$ for the northern summer (Jun-Aug) at (a) Hyy (2002-2006), (b) Bar (2004-2006), (c) Loo (2004-2006), and (d) WBW (1998-1999). Error bars show 1 standard deviation of the range of GPP responses. Data points are split into "diffuse" and "direct" conditions using thresholds of diffuse fractions >80% and <25% to discriminate between the two cases. The two European FluxNet sites, Hyy (panel a) and Loo (c), are needleleaf forests, whereas two North American FluxNet sites, Bar (b) and WBW (d), are broadleaf forests.

Results show that observed and simulated GPP increase with PAR, saturating at high light levels. Further, GPP is consistently higher under diffuse light conditions as expected from the theory of radiative transfer in vegetation canopies. It should be noted, that the sensitivity simulations were performed without tuning important model parameters (eg Vcmax, tree height) to site level conditions and, hence, some discrepancies between model and observations are anticipated. Overall, however, this comparison along with the previous validation studies mentioned in the main ms demonstrate that the model can realistically simulate photosynthetic responses in a range of forest types and light regimes.

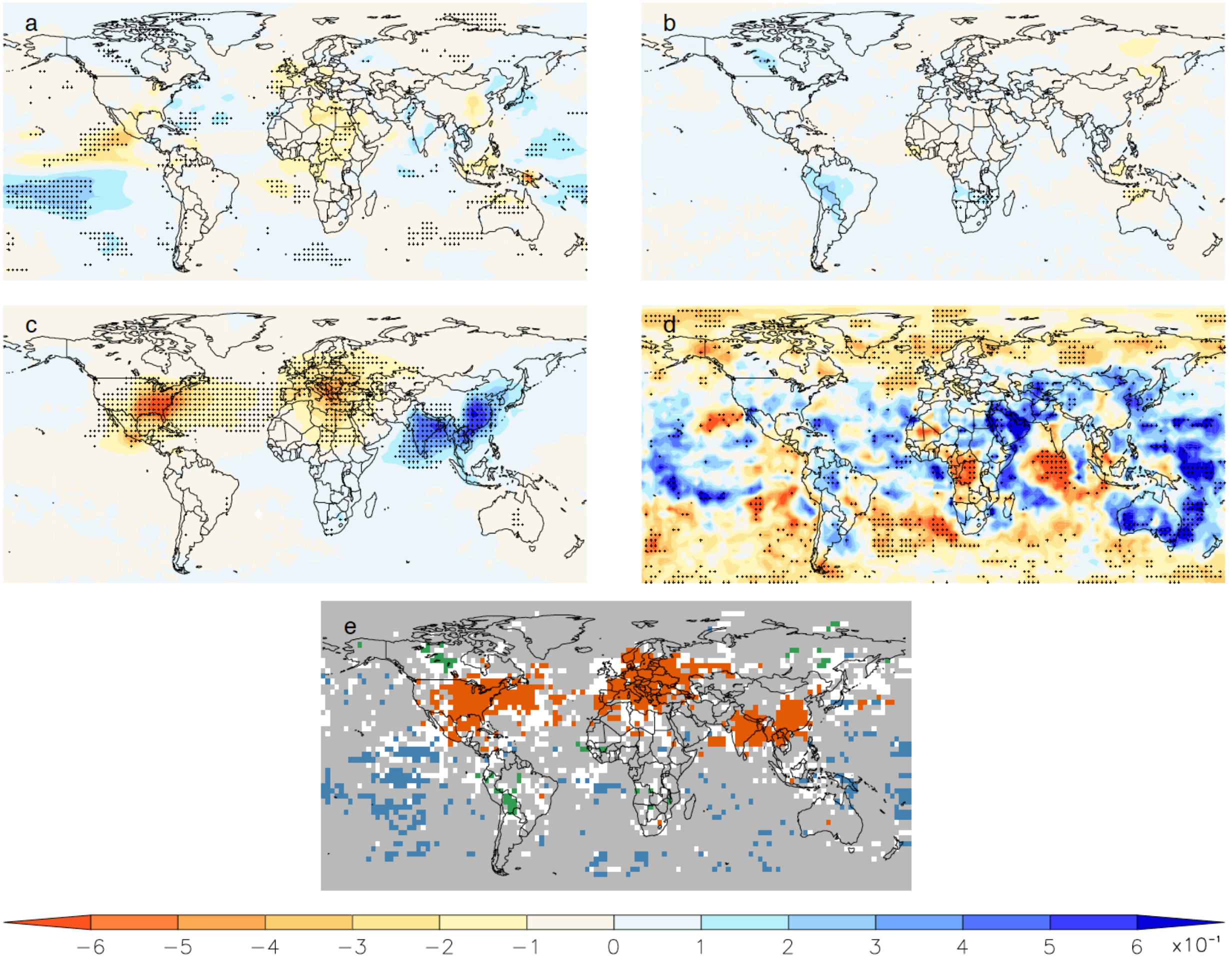


**Figure S7**. Spatial pattern of linear trends ($Wm^{-2}yr^{-1}$) in simulated annual surface diffuse radiation due to each factor (a-d) and the dominating driving factor in the trend (e) over 1998-2010. The isolated factors include (a) climate, (b) fire emissions, (c) fossil fuel emissions, and (d) clouds. Panel (e) shows the dominating driver with climate (blue), fires (green), fossil fuels (orange), and clouds (grey) depicted. White areas depict regions with no factor contributing more than 50% to the total trend (no dominating factor).

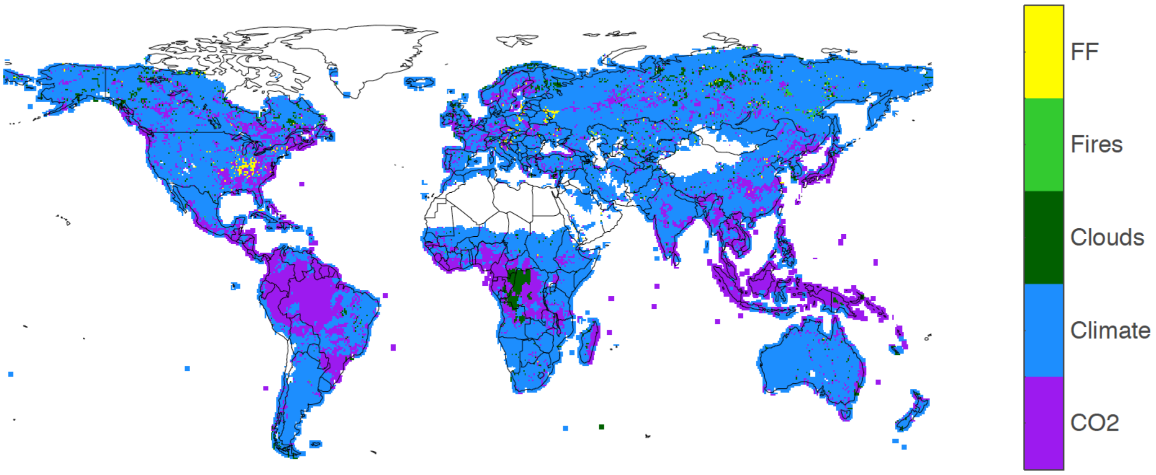


**Figure S8**. Dominant driving factor in simulated (JULES) NPP trend over 1998-2010.

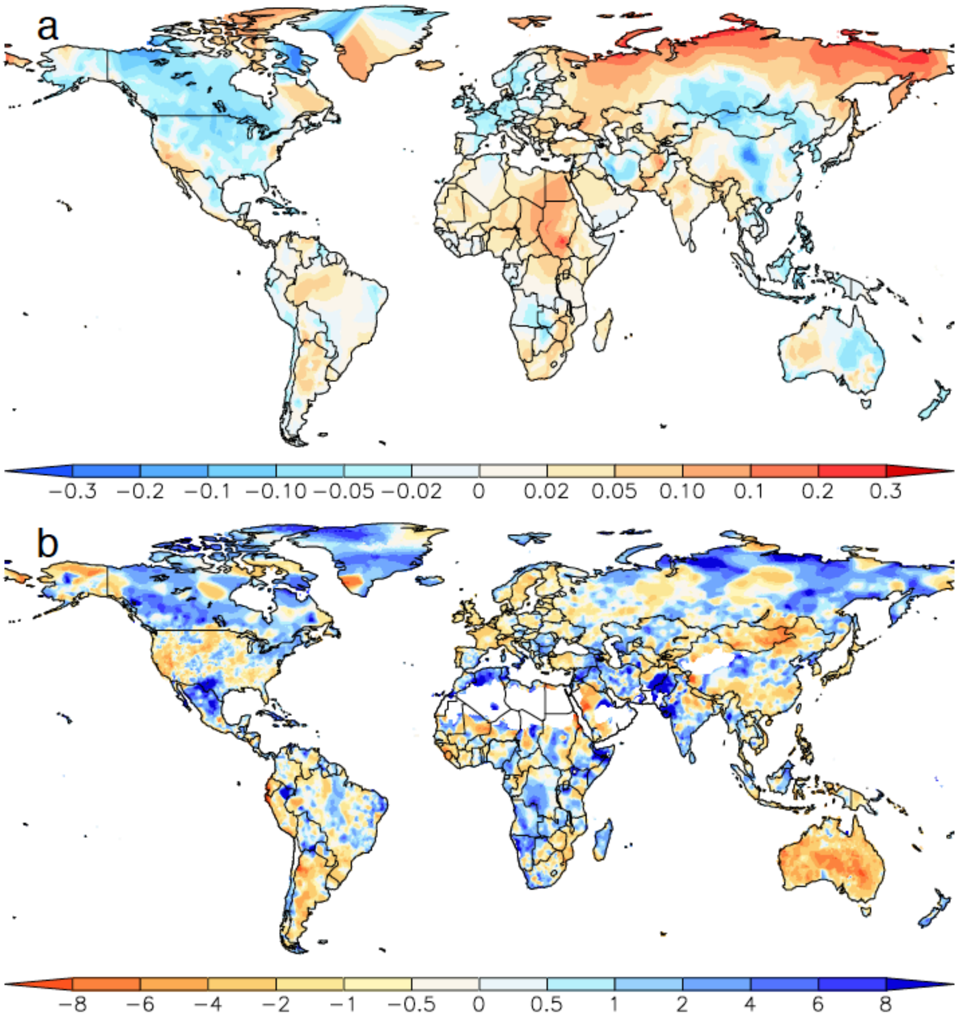


**Figure S9.** Spatial distribution of trends in selected land climate drivers for the period 1998-2010, including (a) temperature ($^{\circ}Cyr^{-1}$) and (b) precipitation ($\%yr^{-1}$).
